# Cases Illustrating the Localization of Valvular Lesions of the Heart

**Published:** 1852-08

**Authors:** Austin Flint


### SELECTIONS

FROM

### FOREIGN AND HOME MEDICAL JOURNALS.

---

*Cases Illustrating the Localization of Valvular Lesions of the Heart.* Reported to the Buffalo Medical Association, by AUSTIN FLINT, M. D.—The study of the auscultatory signs pertaining to heart diseases has, within a few past years, been pursued with such success, that physical exploration in this aspect, is now even more demonstrative and simple than in its application to pulmonary diseases. The physical evidences of heart lesions are, in fact, so reliable, and so readily appreciated, that, in almost every instance, the practitioner is enabled to predicate with positiveness, the existence of the latter upon the presence of the former; and, what is of hardly less consequence, he is able to pronounce with equal confidence on the absence of lesions, in cases in which he fails to discover the characteristic signs. If this remark does not apply to all the lesions to which the heart is subject, it is certainly correct with reference to those seated in the orifices, or valves of this organ. *Vvalular lesions are uniformly accompanied by some of the modifications of the bellows murmur.* If there are exceptions to this rule, they are so few as scarcely to affect our dependence upon it in practice. And, on the other had, the correlative rule follows, that *if the sounds of the heart are perfectly pure, that*

*is to say free from any adventitious sounds, valvular lesions do not exist.*

But physical exploration carries us farther even than this. Not only does it furnish precise information respecting the presence or absence of these lesions, but it enables us to designate, in the great majority of cases, the particular orifice, or orifices at which they are situated. This minuteness of diagnosis, which to those who have devoted no attention to the subject seems almost inconceivable, and appears to be a kind of divination, in reality is attained by very simple means, which a few words will suffice to explain.

In the vast majority of cases valvular lesions are situated in the left side of the heart; that is to say, in the mitral, or aortic orifices. Why this is so, we will not stop to inquire. The fact being as it is, it follows that, for ordinary practical purposes, it suffices to determine whether the lesions are in the mitral or the aortic orifice, or in both these situations.

Lesions thus situated are, as has been stated, uniformly accompanied by a bellows sound. Now, the localization of the lesions involves certain points relating to the bellows sound. These points are two in number. The *first* point relates to the part of the chest at which the bellows sound is heard with its maximum of intensity, and the direction, from this part, in which the sound continues to be heard farthest, and most clearly. If a bellows murmur be developed at the aortic orifice, it will be heard with a maximum of intensity at a part of the chest over this orifice. This part is the intercostal space between the second and third ribs nigh to the sternum. The sound will be heard most distinctly and loudest in this spot It will continue to be heard as the instrument is carried in a direction upward from this part, the sound being transmitted by the walls of the vessels, and the current of blood expelled from the heart. It will be heard farther and louder in this, than in an opposite direction i. e. downward over the body of the organ.

On the other hand, if the bellows murmur be developed at the mitral orifice, the maximum of intensity will be found over the body of the heart, toward the part where the apex strikes against the chest between the fifth and sixth ribs. The sound produced at this orifice is more readily transmitted to the body of the organ, toward the apex, by means of the tendinous chords and fleshy columns, than in an upward direction over the aorta above the heart; consequently, it will be obscure in the latter situation, and rapidly diminish, or disappear, as the instrument is carried above the heart, while it is distinct and loud over the part of the chest lying directly above the organ, and even below this part.

This is a point of distinction which is attended with no real difficulty in its practical application. The principle involved may be illustrated upon the healthy chest. The first sound of the heart is developed, in part, at least, at the mitral orifice. The aortic is passive in the production of this sound. The second sound is due to the semilunar valves of the aorta, and pulmonary artery. Recollecting these facts, let the stethoscope be placed over the chest between the second and third ribs near the sternum. At this point the second sound of the heart will be heard with greater loudness than over the body of the heart; and the sound will be found to be transmitted more readily above than below this spot. The reason for this is the same as in the case of an adventitious sound developed in the same situation.

But the *first* sound will be heard loudest over the body of the body and toward the apex of the heart, obeying the same law which governs the bellows murmur when produced in the same situation.

This experiment can be readily tried.

The bellows murmur, in much the larger proportion of cases, is associated with the first sound of the heart. Now the first sound accompanies the *systole*, and the second the *diastole* of the organ; hence the first is sometimes called the *systolic*, and the second the *diastolic* sound. Recollecting the relations of these two motive acts to the circulation, it will be perceived that when the bellows murmur proceeds from the aortic orifice, it is occasioned by the passage of the blood in the proper direction of the current through this artery. This is not true, however, of the bellows murmur, accompanying the systole, developed at the mitral orifice. The blood passes through this orifice from the auricle to the ventricle, and this takes place during the diastole. A systolic bellows murmur, therefore, if located at the mitral orifice, necessarily involves the existence of lesions of the valves in this situation rendering the orifice patulous, and permitting regurgitation of blood from the ventricle to the auricle, when the heart contracts.

Whenever a bellows murmur accompanies the second, or diastolic sound, it must proceed either from the current of blood in its normal course from the auricule to the ventricle, or from a regurgitating current from the aorta, owing to insufficiency of the valves at this situation. In the latter case, an exception may be furnished to the rule that the sound of a bellows murmur developed at the aortic orifice is more readily transmitted in an upward direction. It is, however, extremely rare that a diastolic bellows murmur is heard; and still rarer, if indeed it be ever true, that a disastolic murmur exists without a systolic. When the former is appreciable, an adventitious sound accompanies both motive acts of the heart, constituting the *see-saw* modification.

The second point involved in the localization of valvular lesions, relates to the *pitch* of the bellows murmur. This point is omitted in some recent works treating of the physical exploration of diseases of the heart. Attention was directed to it especially by Bouillaud, in France, and by Dr. Hope. Why it is excluded from the means of diagnosis by some authors, I am at a loss to understand; for, so far as my opportunities enable me to judge, it is a point of considerable importaace. Dr. Hope doubtless erred in stating that the pitch of sound was affected by distance, but that a distinction in this respect obtains between the sounds produced at the aortic and mitral orifices, is probably true of most cases; and the distinction is easily recognized, especially by one who has what is called a musical ear. The rule as laid down by Hope is, that the mitral bellows murmur has a lower pitch of sound than the aortic. Following the ingenious method suggested by Bouillaud, he represents the diatonic variation in the two sounds by comparing them to a whispered word, on the one hand, and a whispered letter on the other hand. The letter *R.* whispered, will stand as the type of an aortic bellows sound; and the word *who*, of the sound developed at the mitral orifice. On comparing the two sounds produced when this letter and word are whispered, the contrast is sufficiently apparent. These representations are, of course, only approximations to accurrcy, and are applicable to distinguish aortic and mitral bellows sounds only when they are systolic, i. e. accompanying the first sound of the heart.

The valvular lesions are not always limited either to the aortic or mitral orifice. The valves in both orifices may be diseased in the same case. The application of the same principles, however, will in such instances enable us to determine this fact, by showing that a bellows murmur is developed in the two situations.

After this review of the diagnosis, (which has occupied more space than I had intended,) I will invite the attention of the Association to a brief report of three cases that have fallen under my observation within the last few months, in which I have been able to verify, by autopsical examinations, the correctness of the foregoing principles involved in the localization of valvular lesions. In each of the cases the diagnosis was made and recorded before the death of the patient. I have in my possession the hearts of each patient, which I shall exhibit to the Association in connection with a synopsis of the history and physical signs of the cases respectively.

Case 1.—*Disease of mitral valves, allowing regurgitation; with hypertrophy and dilatation. Autopsical appearances.*

J. D., laborer, aged twenty-one. Came under observation October 17, 1851. The following is condensed from the record made at that time:—Had rheumatism when he was sixteen years of age. Several joints were affected in succession, and he went about on crutches for some time. Recollects having had pain in the chest at that time.

Five months ago first observed that in performing labor his breath was short. He was not able to do full day's work, but continued to labor more or less until the first of the present month.

It does not appear that he has had paroxysms of dyspnœa, except when occasioned by exercise, or acts of coughing.

He has had a short hacking cough most of the summer, with very little expectoration.

Appetite has been poor for the last three months. Feels worse after eating, especially after taking animal food; also after drinking spirits.

His aspect does not denote marked anxiety, or suffering. The prolabia are somewhat livid. The alæ dilate considerably with each act of inspiration. He is not emaciated, and the countenance, irrespective of lividity and dilatation of the alæ nasi, is not morbid.

Pulse is 108. Compressible, somewhat jerking.

Skin warm.

Respirations are twenty-eight, and slightly labored.

No œdema of feet or face, and no evidences of ascites.

*Physical Signs.*—Dullness of resonance over increased space in precordia. Considerable heaving of chest. Apex of heart strikes between the sixth and seventh ribs, about three and a half inches below the nipple, on a vertical line running through the nipple.

Above, and over most of the præcordial space the sounds of the heart are normal, *except that the first sound is not so much developed as usual. No bellows sound in these situations. At the apex a distant bellows sound is heard accompanying the first or systolic sound The pitch of the murmur is nearer* WHO *than* R, *but somewhat higher than the former. No bellows murmur appreciable except over apex of heart, and in that situation quite loud.*

In view of the foregoing physical signs, the existence of valvular disease situated in the mitral orifice, permitting regurgitation, was pronounced. The attention of the class then in attendance at the medical college was repeatedly called to the case as illustrating the physical signs denoting localization of lesions in the mitral orifice.

The patient remained under observation until his death, which occurred, January 20, 1852. During the latter part of his life he suffered from dyspnœa, requiring him to remain constantly in a sitting posture. He did not suffer from severe paroxysms of difficult breathing, or orthopnœa. The face presented marked lividity. The pulse was feeble. The impulse in the præcordia became faint and diffused. There was moderate œdema of lower extremities.

*Post-mortem Examination.*—The pericardial sac contained about six ounces of clear serum, with a few flocculi of fibrin. Pericardium transparent and polished. Heart greatly hypertrophied and dilated. Aortic and pulmonary valves free from disease. A few spots of atheroma in the aorta just above the valves. Tricuspid valves free from disease. *Mitral valves thickened, presenting calcareous deposit in considerable quantity, the tendinous cords contracted, occasionally marked insufficiency.* Left auricle greatly dialted, and filled with blood partially coagulated.

Case 2.—*Disease of aortic valves. Dilatation. Autopsical appearances.*

J. *Z.*, German, laborer, aged twenty-eight. Came under observation Oct. 28, 1851. The following is condensed from the record made Oct. 29th:—The patient states that he had good health up to two and a half years ago. He was then seized, suddenly, with severe pain in both hip joints, which continued more or less until last Christmas. About Christmas an abscess discharged on the inner part of thigh, eight or ten inches below Poupart's ligament. The discharge has continued to the present time, and still continues. He is able to use both hip joints freely.

In May last he first noticed that he was short winded. Previous to that time he was able to labor several days at a time. Shortly afterward he was obliged to give up work. He experienced at this time, occasionally, pain in the precordial region, but has had none within the last month.

He is too feeble to give a very full previous history.

He lies somnolent, in a semi erect posture, but is easily roused.

Respirations forty-eight, and labored.

Aspect notably pallid, with slight œdema of face. Feet and legs considerably œdematous.

Abdomen meteorized, without the physical evidences of ascites.

Skin cold.

Pulse 108, very small and feeble.

Urine deposits considerable albumen on testing with heat and nitric acid.

*Physical Signs.*—Flatness on percussion over a space in precordia five or six inches transversely across the nipple. Point of impulse of heart felt about an inch below the nipple, and to the left of a vertical line passing through the nipple. No heaving of the chest. Sounds of heart distant and confused. *A bellows murmur, high in pitch, (as high as* R.,*) accompanies the first sound, heard between*

*second and third ribs nigh to sternum, and not over the body or at apex of heart.*

The diagnosis noted in connection with the record from which the foregoing is taken was, *lesion of aortic valves; increased effusion within pericordial sac, with dilatation.*

The attention of the medical class was called to this case as illustrating the localization of valvular lesions, by physical signs, in the aortic orifice.

Death occurred, in this case, on the 29th October.

*Post-mortem Appearances.*—Left ventricle and auricle filled with grumous blood, and a few white coagula. Left ventricle dilated to about twice its normal capacity. Thickness of walls but little increased. *All the valves free from disease except those at the aortic orifice, which were studded with vegetations and calcareous deposit.* Considerable atheromatous deposit in aorta above the valves. Effusion within pericardial sac estimated to be between four or five ounces.

Lungs healthy, and not greatly congested.

Liver not diminished in volume. The interlobular veins deeply congested, occasioning a granular appearance of the lobules.

Kidneys congested.

Other organs not examined.

Case 3.—*Disease of aoriic valves. Great hypertrophy. Angina pectoris. Autopsical appearances.*

Mr. R. applied to me July, 1851, aged about forty. The following is condensed from the record made July 17th:—Patient was attacked three years ago with severe pain in præcordial region. He was riding at the time, and the pain obliged him to lie down at once. Had always previous enjoyed excellent health. Had never had rheumatism. Since that time he has been subject to repetitions of similar attacks of intense pain in præcordial and sternal regions, shooting down into both upper extremities. The attacks are manifestly those of angina. They occur, more or less, every week, and, of late, once, and sometimes twice daily. The pain is excruciating. It is accompanied and followed by palpitations, and by great dyspnœa. Attacks are brought on by exercise, but they frequently occur without any exciting cause. He has been unable to labor for the past three years. He is thin, but aspect not morbid. Appetite good, bowels regular.

There is no œdema. He is not habitually short-winded. His sources of complaint are the paroxysms described. If these were relieved he thinks he should be well. He has consulted several physicians of the city, and submitted to repeated bleedings with no good effect. He has also had the advice of various empirics.

*A loud bellows murmur is developed in the aortic orifice, the pitch as high as* R, *the maximum above the heart, accompanying the first sound.*

Pulse well developed, not irregular. He states that the pulse intermits during the paroxysms sometimes, and sometimes not.

The dorsal spine is tender on pressure.

*Diagnosis. Valvular disease of aortic orifice.*

The following prescription was made at this time:

℞. Ext. Hyosciam, ℈i.

Tinct. Digitalis, f℥i.

Aquæ, ℥ii.

Teaspoonful three times daily.

Croton oil to be applied to spine.

August 11th. The following is noted:—This patient called and states that he has had no paroxysms of pain since taking the above prescription until last evening, and the suffering then was not so severe as usual. He has taken the medicine until four or five days since. It was then omitted because his supply was exhausted. Two or three days after commencing its use he called to complain of not being able to see minute objects. He was unable, for example, to see letters distinctly enough to read. The pupils were dilated. He diminished the dose of the medicine, and the affection of vision passed off in a few days. The medicine evidently produces slowness and intermittency of pulse. He has made the back sore with the croton oil.

November 10th. This patient called to see me about two weeks since, and stated that he suffered nightly from paroxysms of intolerable pain, with dyspnœa. He was sure that he could not live long, and desired that his body should be examined after death He died on the morning of this date, and the chest was examined late in the forenoon of the same day.

The heart was greatly hypertrophied, being more than double the normal size. The enlargement was most marked in the left ventricle. The ventricular walls were but slightly increased in thickness. Aorta enlarged for several inches above the heart, and the lining membrane opaque, and roughened by atheromatous deposit, which in several points presented calcareous degeneration. *Aortic valves thickened and much contracted.* Pulmonary valves healthy. Mitral valves slightly thickened, but not diseased sufficiently to compromise their functions.

Lungs healthy.

Other organs not examined.—*Buffalo Medical Journal.*

